# Simultaneous *Brg1* Knockout and *MYCN* Overexpression in Cerebellar Granule Neuron Precursors Is Insufficient to Drive Tumor Formation but Temporarily Enhances their Proliferation and Delays their Migration

**DOI:** 10.1007/s12311-020-01219-2

**Published:** 2021-01-02

**Authors:** Dörthe Holdhof, Ji Hoon On, Melanie Schoof, Carolin Göbel, Ulrich Schüller

**Affiliations:** 1 Department of Pediatric Hematology and Oncology, University Medical Center Hamburg-Eppendorf, Hamburg, Germany; 2 Research Institute Children’s Cancer Center Hamburg, Martinistrasse 52, N63 (HPI), D-20251 Hamburg, Germany; 3 Institute of Neuropathology, University Medical Center Hamburg-Eppendorf, Hamburg, Germany

**Keywords:** Medulloblastoma, BRG1, MYCN, Mouse, Migration deficit

## Abstract

Medulloblastoma (MB) is the most common malignant brain tumor in childhood. According to the World Health Organization (WHO) classification of central nervous system (CNS) tumors, this embryonal tumor is divided into a wingless (WNT)-activated, Sonic hedgehog (SHH)-activated, and non-WNT/non-SHH entity. The latter is poorly defined but frequently carries mutations in *Brahma-related gene 1* (*BRG1*) or amplifications of *MYCN*. Here, we investigated whether a combination of a *Brg1* knockout and an overexpression of *MYCN* in cerebellar granule neuron precursors or multipotent neural stem cells is sufficient to drive brain tumor formation in mice. To this end, we generated *Math1-creER*^*T2*^*::Brg1*^*fl/fl*^*::lslMYCN* and *hGFAP-cre::Brg1*^*fl/fl*^*::lslMYCN* mice, respectively. We did not observe brain tumor formation in any of these models. *hGFAP-cre::Brg1*^*fl/fl*^*::lslMYCN* mice revealed severe CNS abnormalities with short survival, similar to the situation with a sole loss of *Brg1*, as we previously described. Investigation of *Math1-creER*^*T2*^*::Brg1*^*fl/fl*^*::lslMYCN* mice with a tamoxifen induction at postnatal day 3 revealed a regular survival but significant increase in cerebellar granule neuron precursor proliferation, followed by a delayed inward migration of these cells. This is in stark contrast to the hypoplastic cerebellum that we previously observed after embryonic deletion of *Brg1* in *Math1* positive cerebellar granule neurons. Our results indicate a time-specific function of *Brg1* in cerebellar granule neuron precursors. Yet, the exact temporal and spatial origin of non-WNT/non-SHH MB remains unclear.

**Supplementary Information:**

The online version contains supplementary material available at 10.1007/s12311-020-01219-2.

## Introduction

Medulloblastoma (MB) is the most common malignant brain tumor in children [1]. This embryonal tumor arises in the *posterior fossa* and can be divided into at least three molecular subgroups: wingless (WNT)-activated MB, Sonic hedgehog (SHH)-activated MB, and non-WNT/non-SHH MB [2]. WNT and SHH MBs are characterized by activating mutations in the respective pathways, but driver mutations in non-WNT/non-SHH, which can be further segregated into Group 3 and Group 4 MBs, are less well understood [3]. A number of large-scale sequencing studies revealed that missense mutations in the epigenetic modifier *Brahma-related gene 1* (*BRG1*, *SMARCA4*, *BAF190A*) are found in 4.3–8.8% of all MBs, but especially in WNT and Group 3 MBs [4–6]. In the study performed by Jones et al., *BRG1* was the most common mutated gene in Group 3 MB. *BRG1* encodes one of the two mutually exclusive ATPase subunits of the SWItch/sucrose nonfermenting (SWI/SNF) chromatin remodeling complex, which regulates gene expression by increasing nucleosome mobility [7–9]. It has been described as a tumor suppressor in different cancer entities, such as rhabdoid tumors, small cell carcinoma of the ovary, hypercalcemic type (SCCOHT), and lung cancer [10–14]. Still, a *Brg1* knockout in various different neural stem cell (NSC) populations in mice was not sufficient to drive tumor development but caused hypoplasia of diverse brain regions [15–17]. Amplifications of the proto-oncogene *MYCN* have been identified in several cancer entities, including tumors of the central nervous system (CNS) such as high-grade gliomas, spinal ependymoma and MB [18–21]. In MB, they are found in SHH, Group 3 and Group 4 MBs and are associated with a poor outcome [4–6, 19, 22]. MYCN is a transcription factor of the MYC family and is important for cell growth, apoptosis, tumor cell metabolism and normal cerebellar development [21, 23, 24]. Swartling et al. demonstrated that overexpression of *MYCN* in Glutamate transporter 1 (Glt1) positive cells gives rise to murine MB [25]. However, these tumors occurred rather late in development and accumulated sporadic *p53* mutations in addition to the *MYCN* alteration [26]. Furthermore, due to the broad expression of the promoter and the late tumor detection, the exact anatomical origin of these tumors remains obscure. The same group recently reported that ectopic expression of *MYCN* in human induced pluripotent stem cell-derived neuroepithelial stem (NES) cells increases proliferation in vitro. Orthotopic transplantation of these NES cells into mice results in tumor formation resembling human SHH MB, again without precise information about the tumor origin [27]. In *hGFAP* expressing cells, *MYCN* amplification did not result in MB formation, possibly due to the lack of a second hit [28]. In this study, we were interested whether *Brg1* knockout and *MYCN* overexpression might have synergistic effects in the development of tumors in the *posterior fossa*. Therefore, we generated *Math1-creER*^*T2*^*::Brg1*^*fl/fl*^*::lslMYCN*^*fl/fl*^ *(Mert::B*^*fl/fl*^*::N*^*fl/fl*^*)* and *hGFAP-cre::Brg1*^*fl/fl*^*::lslMYCN*^*fl/fl*^ *(hG::B*^*fl/fl*^*::N*^*fl/fl*^*)* mice to investigate the combination in postnatal granule neuron precursor cells (GNPCs) and multipotent NSCs, respectively [29, 30]. Mice carrying the inducible *creER*^*T2*^ transgene under the control of the *Math1* promoter received tamoxifen at postnatal day 3 (P3) to induce the cre recombinase. In *hG::B*^*fl/fl*^*::N*^*fl/fl*^ mice, the cre recombinase was constitutively active from embryonal day 13.5 (E13.5) onwards [30]. We observed that the loss of *Brg1* in combination with an additional overexpression of *MYCN* in *Math1* expressing cells resulted in changes regarding cell survival parameters in the external granular layer (EGL) of the cerebellum. Furthermore, GNPCs showed delayed inward migration. However, the adult cerebellum appeared normal with regular organization into inner granular layer (IGL), Purkinje cell layer (PCL) and molecular layer (ML) and without any signs of tumor development. Amplification of *MYCN* in addition to the loss of *Brg1* in *hGFAP* expressing cells did not rescue the phenotype caused by the deprivation of *Brg1* alone. As we published earlier, *Brg1* deficiency in *hGFAP* positive cells causes a hypoplastic cerebellum [17]. Likewise, *hG::B*^*fl/fl*^*::N*^*fl/wt*^, and *hG::B*^*fl/fl*^*::N*^*fl/fl*^ mice presented with an underdeveloped cerebellum without organization into lobuli and with absence of the characteristic cerebellar layering. They died after about 2 weeks without any signs of tumor formation. Hence, we conclude that *MYCN* amplification and *Brg1* deficiency disturb cerebellar development but are not sufficient to drive brain tumors originating from *hGFAP* or *Math1* positive precursor cells.

## Material and Methods

### Mice

*hGFAP-cre* (JAX #004600) and *Math1-creER*^*T2*^ (JAX #7684) mice were purchased from The Jackson Laboratory [30, 31]. *Brg1*^*fl/fl*^ and *lox-STOP-lox-MYCN*^*fl/fl*^ (*lslMYCN*^*fl/fl*^) mice have previously been generated and described [32, 33]. These mice were crossed to generate *Math1-creER*^*T2*^*::Brg1*^*fl/wt*^ *(Mert::B*^*fl/wt*^*)*, *Math1-creER*^*T2*^*::Brg1*^*fl/fl*^ *(Mert::B*^*fl/fl*^*)*, *Math1-creER*^*T2*^*::Brg1*^*fl/fl*^*::lslMYCN*^*fl/wt*^ (*Mert::B*^*fl/fl*^*::N*^*fl/wt*^), *Math1-creER*^*T2*^*::Brg1*^*fl/fl*^*::lslMYCN*^*fl/fl*^ (*Mert::B*^*fl/fl*^*::N*^*fl/fl*^), *hGFAP-cre::Brg1*^*fl/wt*^*::lslMYCN*^*fl/wt*^ (*hG::B*^*fl/wt*^*::N*^*fl/wt*^), *hGFAP-cre::Brg1*^*fl/wt*^*::lslMYCN*^*fl/fl*^ (*hG::B*^*fl/wt*^*::N*^*fl/fl*^), *hGFAP-cre::Brg1*^*fl/fl*^*::lslMYCN*^*fl/wt*^ (*hG::B*^*flfl*^*::N*^*fl/wt*^), and *hGFAP-cre::Brg1*^*fl/fl*^*::lslMYCN*^*fl/fl*^ (*hG::B*^*flfl*^*::N*^*fl/fl*^) mice. They were kept on a C57Bl/6J background. Mice carrying the *Math1-creER*^*T2*^ knock-in and respective controls were treated with a single dose of 0.6 mg tamoxifen (Sigma-Aldrich) dissolved in corn oil at P3 via intraperitoneally (i.p.) injections. To label proliferating cells in vivo, 5-bromo-2-deoxyuridine (BrdU, Sigma-Aldrich) was injected i.p. (25 μg/g bodyweight) 2 h prior to sacrifice. Genotyping of genomic DNA from mouse ear biopsies or tail tips was performed by PCR. Mice were kept on a 12 h dark/light cycle; water and food were available ad libitum. Animals of both sexes were used for the experiments. All experimental procedures were approved by the Government of Hamburg, Germany (113/16).

### Immunohistochemistry

For hematoxylin and eosin (H&E) and immunohistochemistry (IHC) stains, brain tissue was fixed in 4% paraformaldehyde/PBS for at least 12 h. The tissue was dehydrated, embedded in paraffin, and sectioned at 4 μm according to standard protocols. All IHC stains were performed on a Ventana System (Roche) using standard protocols. The following antibodies were used: mouse anti-BrdU (Invitrogen, clone MoBU-1, #B35128, 1:100), rabbit anti-Brg1 (Abcam, ab110641, 1:200), rabbit anti-cleaved Caspase 3 (cl. Casp3; Asp175; Cell Signaling Technology, #9664, 1:100), rabbit anti-MycN (Cell Signaling, #51705, 1:100), mouse anti-Pax6 (DSHB, 1:50), mouse anti-phospho-Histone H3 (pHH3, Cell Signaling Technology, #9706, 1:200), rabbit anti-S100 (DAKO, Z0311, 1:100).

### Image Quantifications and Statistical Analysis

In order to quantify the proportion of cells positive for a specific marker, we used IHC stained sagittal sections and counted their numbers in relation to the total number of cells per field using the ImageJ software (Wayne Rasband, National Institute of Health, USA). For marker expression in the EGL, we quantified the cells in the EGL between lobuli V and VI. To analyze the proportion of Pax6 positive cells in the ML, we counted the cells in lobulus VI. Marker expression was quantified for at least three animals per genotype by a blinded investigator. All statistical analyses were performed using *Prism Software Version 7* (GraphPad Software, Inc., San Diego, USA). For quantifications of marker expression in the EGL and the ML, a Tukey’s multiple comparisons test and for survival analyses, a log-rank Mantel-Cox test was performed.

## Results

### A Postnatal Loss of *Brg1* With or Without Additional Amplification of *MYCN* in *Math1* Expressing Cells Causes an Increase in Proliferation

**Fig. 1:**
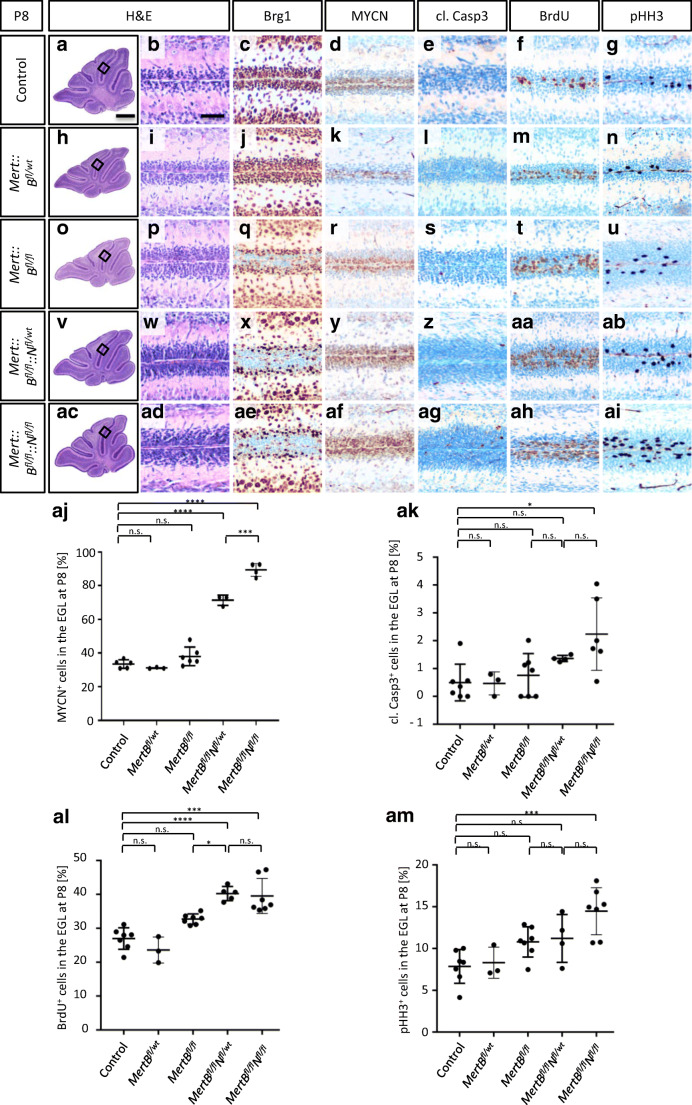
*Mert::B*^*fl/fl*^*::N*^*fl/wt*^ and *Mert::B*^*fl/fl*^*::N*^*fl/fl*^ mice display significant alterations in the expression of markers for apoptosis and cell proliferation in the external granule layer. Representative sagittal H&E sections of mouse cerebella are shown at P8 (**a**, **b**, **h**, **i**, **o**, **p**, **v**, **w**, **ac**, **ad**). There are no morphological alterations in the EGL in any of the mutants compared to controls. Brg1 knockout and MYCN amplification are confirmed by IHC (c, d, j, k, q, r, x, y, ae, af). Apoptosis induction is indicated by cl. Casp3 stainings (**e**, **l**, **s**, **z**, **ag**). Proliferating cells are identified by BrdU and pHH3 stainings (f, g, m, n, t, u, aa, ab, ah, ai). Quantifications of MYCN, cl. Casp3, BrdU and pHH3 positive cells are shown in aj, ak, al and am, respectively. The control group includes *B*^*fl/fl*^, *B*^*fl/wt*^, *B*^*fl/fl*^*::N*^*fl/wt*^ and *B*^*fl/fl*^*::N*^*fl/fl*^ mice (*n* = 7). The mutant groups are *Mert::B*^*fl/wt*^ (*n* = 3), *Mert::B*^*fl/fl*^ (*n* = 7), *Mert::B*^*fl/fl*^*::N*^*fl/wt*^ (*n* = 5), and *Mert::B*^*fl/fl*^*::N*^*fl/fl*^ (n = 7). The scale bar in A corresponds to 400 μm and is representative for h, o, v and ac. The scale bar in B corresponds to 50 μm and is representative for all other panels. *p < 0.05, **p < 0.01, ***p < 0.001, ****p < 0.0001. n.s., not significant

A prenatal loss of *Brg1* in *Math1* positive cells results in cerebellar hypoplasia [16]. Therefore, we chose the tamoxifen-inducible creER^T2^ recombinase to generate mice with a postnatal *Brg1* loss either alone or with additional overexpression of the human *MYCN* in *Math1* expressing cells. We injected tamoxifen at P3 to activate cre-mediated recombination. In a first approach, we were interested in the short-term effects and sacrificed the animals 5 days after tamoxifen injections at P8. At this time, proliferation of GNPCs peaks during normal murine cerebellar development [34, 35]. H&E stains revealed no morphological alterations at P8 as animals of all genotypes had normal cerebella with regular lobuli and distinct layers (Fig. 1a, b, h, i, o, p, v, w, ac, ad). We further investigated the extent of Brg1 loss and MYCN overexpression by IHC. In controls and in *Mert::B*^*fl/wt*^ animals, all cells were positive for Brg1 and MYCN was expressed in the outer EGL (Fig. 1c, d, j, k). The EGL of the remaining three genotypes consisted of Brg1 negative cells in the outer EGL and Brg1 positive cells in the inner EGL (Fig. 1q, x, ae). This result was expected, since we induced Brg1 loss by tamoxifen treatment not before P3. The number of MYCN expressing cells was significantly increased in the presence of the *lslMYCN* transgenes (71% and 89% in *Mert::B*^*fl/fl*^*::N*^*fl/wt*^ and *Mert::B*^*fl/fl*^*::N*^*fl/fl*^, respectively) compared to controls (33%), *Mert::B*^*fl/wt*^ (31%) and *Mert::B*^*fl/fl*^ (38%) mice (Fig. 1y, af, aj).

Next, we investigated the influence of *Brg1* loss and *MYCN* overexpression on cell survival by examining apoptosis and proliferation marker expression. We stained for cl. Casp3 as an indicator for apoptosis induction (Fig. 1e, l, s, z, ag, ak). Quantification of cl. Casp3 stainings revealed no significant differences in the number of apoptotic cells in the EGL of control mice (0.5%) and mice with both a hetero- (0.47%) and a homozygous loss of *Brg1* (0.76%). However, in *Mert::B*^*fl/fl*^*::N*^*fl/fl*^ mice, but not in *Mert::B*^*fl/fl*^*::N*^*fl/wt*^ mice, the additional overexpression of *MYCN* caused a significant increase in apoptosis in the EGL as 2.24% and 1.36% of cells were cl. Casp3 positive, respectively (Fig. 1ak). In order to investigate proliferation, we pulsed the animals 2 h prior to sacrifice with BrdU, a thymidine analog that is incorporated into newly synthesized DNA during the S phase of the cell cycle. We stained for BrdU and additionally for pHH3 to examine two independent proliferation markers (Fig. 1f, g, m, n, t, u, aa, ab, ah, ai). There were no significant differences in the number of BrdU positive cells in the EGL of control (27.0%), *Mert::B*^*fl/wt*^ (23.6%) and *Mert::B*^*fl/fl*^ mice (32.7%), even though there was a trend towards more proliferating cells in the latter (Fig. 1al). However, in case of a simultaneous *MYCN* amplification, there were significantly more BrdU positive cells in the EGL. In *Mert::B*^*fl/fl*^*::N*^*fl/wt*^ mice, 40.2% of cells and in *Mert::B*^*fl/fl*^*::N*^*fl/fl*^ mice, 39.5% of cells had incorporated BrdU during S phase (Fig. 1al). Likewise, the percentage of pHH3 expressing cells was insignificantly different in case of a Brg1 knockout without *MYCN* amplification (Fig. 1am). In the EGL of controls, *Mert::B*^*fl/wt*^ mice and *Mert::B*^*fl/fl*^ mice, 7.86%, 9.42%, and 10.8% were pHH3 positive, respectively. Again, these results indicate that a *Brg1* deficiency caused a trend towards more proliferating cells. Amplification of *MYCN* caused a significant rise in the number of pHH3 expressing cells in the EGL of *Mert::B*^*fl/fl*^*::N*^*fl/fl*^ mice (14.5%), but not of *Mert::B*^*fl/fl*^*::N*^*fl/wt*^ mice (11.2%).

Taken together, the combination of a *Brg1* deficiency with *MYCN* overexpression significantly alters the expression of cell survival parameters in the EGL at P8.

### *Brg1* Deficiency and *MYCN* Amplification Decreases Inward Migration of Granule Neurons

**Fig. 2:**
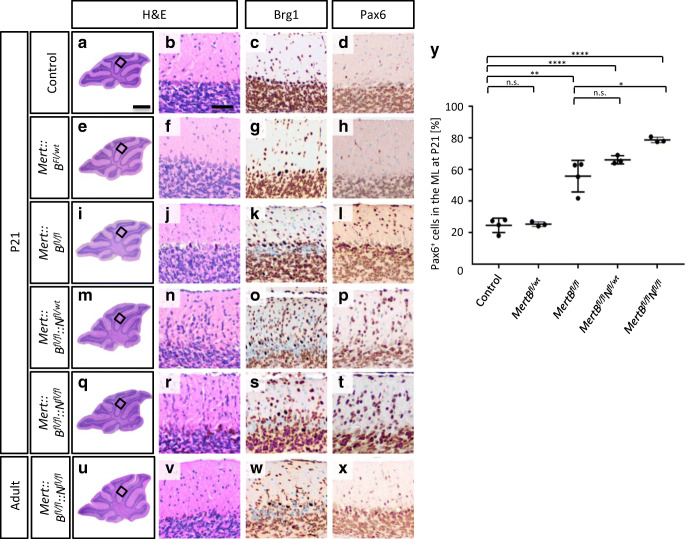
*Mert::B*^*fl/fl*^*::N*^*fl/wt*^ and *Mert::B*^*fl/fl*^*::N*^*fl/fl*^ mice display increased numbers of Pax6 positive cells in the molecular layer. Representative sagittal H&E sections of whole cerebella are shown at P21 and at adult age (a, e, i, m, q and u). Brg1 knockout (c, g, k, o, s, w) is examined by IHC. Sagittal H&E (b, f, j, n, r, v) and Pax6 (d, h, l, p, t, x) stainings of the EGL and IGL display an aggregation of Brg1 deficient granule cells. Quantification of Pax6 expressing cells in the ML of animals at P21 are shown in Y. The control group includes *B*^*fl/fl*^*::N*^*fl/wt*^ and *B*^*fl/fl*^*::N*^*fl/fl*^ mice (*n* = 4). The mutant groups (*Mert::B*^*fl/wt*^, *Mert::B*^*fl/fl*^, *Mert::B*^*fl/fl*^*::N*^*fl/wt*^ and *Mert::B*^*fl/fl*^*::N*^*fl/fl*^) include 3 animals each. The scale bar in A corresponds to 400 μm and is representative for e, i, m, q, and u. The scale bar in B corresponds to 50 μm and is representative for all other panels. **p < 0.01, ***p < 0.001, ****p < 0.0001. n.s., not significant

In mice, cerebellar cortical development is finalized at an age of 3 weeks resulting in three distinct layers: ML, PCL and IGL [36]. In order to analyze how the postnatal knockout of *Brg1* with or without simultaneous overexpression of *MYCN* influenced the ontogenesis of the cerebellum, we sacrificed the mice at P21 and investigated the morphology by H&E stains (Fig. 2a, b, e, f, i, j, m, n, q, r). In *Mert::B*^*fl/fl*^ mice, it appeared as if more cells were present in the ML compared to control and *Mert::B*^*fl/wt*^ mice. This effect seemed to be even more pronounced in *Mert::B*^*fl/fl*^*::N*^*fl/wt*^ mice and *Mert::B*^*fl/fl*^*::N*^*fl/fl*^ mice. To validate, whether the supernumerary cells in the ML were Brg1 deficient, we stained for Brg1 (Fig. 2c, g, k, o, s). In all mice not carrying a homozygously floxed Brg1 allele, Brg1 was expressed in all cells. In all three mutants, Brg1 negative cells were not only observed in the IGL, but also in the ML. This suggested that *Brg1* loss resulted in decelerated migration of GNPCs from the EGL to the IGL. In order to verify that these cells were GNPCs, we stained for Pax6 and quantified the proportion of Pax6 expressing cells in the ML (Fig. 2d, h, l, p, t, y). In both, the ML of controls and of *Mert::B*^*fl/wt*^ mice, 24.6% and 25.4% of cells were Pax6 positive. In *Mert::B*^*fl/fl*^, *Mert::B*^*fl/fl*^*::N*^*fl/wt*^, and *Mert::B*^*fl/fl*^*::N*^*fl/fl*^ mice, there were significantly more GNPCs in the ML, as indicated by Pax6 positivity (55.8%, 66.2%, and 78.7%, respectively).

In order to validate how the changes in apoptosis and proliferation of GNPCs at P8 and the alterations in the GNPCs’ migratory behavior affected the morphology of the adult cerebellum, we examined the brains of 6-month-old mice. Similar to *Mert::B*^*fl/wt*^, *Mert::B*^*fl/fl*^, and *Mert::B*^*fl/fl*^*::N*^*fl/wt*^ mice (data not shown), H&E analysis of cerebella from *Mert::B*^*fl/fl*^*::N*^*fl/fl*^ mice revealed that the overall morphology appeared normal (Fig. 2u–x). Brg1 negative cells were found in both, the ML and the IGL, and only a very small fraction of Pax6 expressing cells was present in the ML, similar to the expression profile of control P21 cerebella. We therefore conclude that GNPC inward migration is delayed after loss of *Brg1* and overexpression of *MYCN* but is completed at the age of 6 months. In order to exclude tumor growth in a small proportion of such animals, we observed a total of 14 *Mert::B*^*fl/fl*^*::N*^*fl/wt*^ and 12 *Mert::B*^*fl/fl*^*::N*^*fl/fl*^ animals over a period of 6 months (Supplementary Fig. 1a). Three and two animals of these cohorts died, respectively, without any preceding symptoms. Therefore, we were not able to analyze their brains or bodies. All other animals underwent full autopsy after 6 months, but we did not detect any tumor growth or other abnormalities. Although we cannot report on the autopsy of 5 out of 25 animals, we assume that a simultaneous loss of *Brg1* and overexpression of *MYCN* in *Math1* positive GNPCs is not sufficient to drive tumor growth.

### Amplification of *MYCN* in Multipotent NSCs Does Not Rescue the Effects Caused by the Loss of *Brg1*

**Fig. 3:**
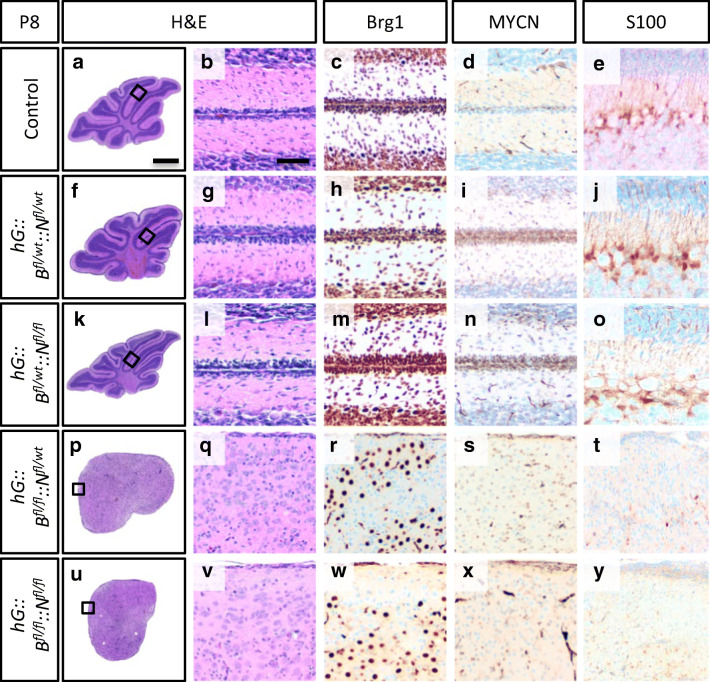
*hG::B*^*fl/fl*^*::N*^*fl/wt*^ and *hG::B*^*fl/fl*^*::N*^*fl/fl*^ mice display a hypoplastic cerebellum. Representative sagittal H&E sections of mouse cerebella at P8 indicate that compared to controls (**a**, **b**) a heterozygous *Brg1* knockout has no morphological consequences (**f**, **g**, **k**, **l**), whereas a homozygous *Brg1* loss leads to a hypoplastic cerebellum (**p**, **q**, **u**, **v**). Panels **c**, **h**, **m**, **r** and **w** show Brg1 stains and **d**, **i**, **n**, **s**, and **x** MYCN stains. S100 stains are presented in panels **e**, **j**, **o**, **t**, **y**. The control group includes *B*^*fl/fl*^*::N*^*fl/wt*^ and *B*^*fl/fl*^*::N*^*fl/fl*^ mice (*n* = 5). The mutant groups include *hG::B*^*fl/wt*^*::N*^*fl/wt*^ (*n* = 4), *hG::B*^*fl/wt*^*::N*^*fl/fl*^ (*n* = 3), *hG::B*^*fl/fl*^*::N*^*fl/wt*^ (*n* = 4), and *hG::B*^*fl/fl*^*::N*^*fl/fl*^ (*n* = 4). The scale bar in A corresponds to 400 μm in **a**, **f**, **k**, **p**, and **u** and the scale bar in B corresponds to 50 μm and is representative for all other panels

In order to examine the combination of *Brg1* deficiency and *MYCN* amplification in a spatially and temporally different setting, we generated *hG::B::N* mice. In contrast to the induced cre recombinase activity in postnatal GNCPs in *Mert::B::N* animals, *hGFAP* drives the constitutive expression of the cre recombinase in multipotent NSCs from E13.5 onwards. Consequently, *Brg1* loss and *MYCN* overexpression can be observed in almost all cerebellar cells, except for Purkinje cells and choroid plexus epithelium [30]. We previously reported that multipotent NSCs marked by *hGFAP* expression depend on the proper expression of *Brg1* to give rise to all major brain regions [17]. For instance, the cerebella of *hGFAP-cre::Brg1*^*fl/fl*^ mice are hypoplastic without foliation or lamination. In the present study, we investigated, whether an additional *MYCN* amplification was able to rescue this phenotype or would even give rise to brain tumors. For this purpose, we generated *hG::B*^*fl/wt*^*::N*^*fl/wt*^, *hG::B*^*fl/wt*^*::N*^*fl/fl*^, *hG::B*^*flfl*^*::N*^*fl/wt*^ and *hG::B*^*flfl*^*::N*^*fl/fl*^ mice. In case of a heterozygous *Brg1* deficiency, the cerebella at P8 appeared normal in H&E stains compared to controls (Fig. 3a, b, f, g, k, l). As expected, Brg1 was detectable in all cells of these mice (Fig. 3c, h, m), whereas the number of MYCN positive cells seemed increased in the EGL of *hG::B*^*fl/wt*^*::N*^*fl/wt*^ and *hG::B*^*fl/wt*^*::N*^*fl/fl*^ mice (Fig. 3d, i, n)In contrast, the cerebella of *hG::B*^*flfl*^*. ::N*^*fl/wt*^ and *hG::B*^*flfl*^*::N*^*fl/fl*^ mice were severely underdeveloped and resembled those of *hGFAP-cre:.Brg1*^*fl/fl*^ mice. Neither lobules nor distinct layers were detectable in H&E stains (Fig. 3p, q, u, v). Like in *hGFAP-cre::Brg1*^*fl/fl*^ mice, Brg1 expression was lost in a fraction of cells distributed across the entire cerebellum of *hG::B*^*flfl*^*::N*^*fl/wt*^ and *hG::B*^*flfl*^*::N*^*fl/fl*^ mice (Fig. 3r, w). As the *hGFAP* promoter is expressed in the majority of cerebellar cells or their ancestors [30], Brg1 deficiency was likely to be present in most cell types. However, only a small proportion of cells were positive for MYCN (Fig. 3s, x). This indicates either that the recombination of two floxed transgenes was not efficient or that especially those cells with enhanced *MYCN* expression were depleted from the developing brain until P8.

Next, we examined, whether the absence of distinct layers in *hG::B*^*flfl*^*::N*^*fl/wt*^ and *hG::B*^*flfl*^*::N*^*fl/fl*^ cerebella was caused by impaired neuronal migration due to altered Bergmann glia cell morphology. Proper neuronal migration is highly dependent on Bergmann glia cells [37], and *hGFAP*-mediated cre expression is present in these cells [30]. We employed S100 as a Bergmann glia marker [38] that stains the entire cells including their radial processes. In controls, *hG::B*^*fl/wt*^*::N*^*fl/wt*^ and *hG::B*^*fl/wt*^*::N*^*fl/fl*^ mice, Bergmann glia cells were located in the PCL and their processes extended to the pial surface (Fig. 3e, j, o). In *hG::B*^*flfl*^*::N*^*fl/wt*^ and *hG::B*^*flfl*^*::N*^*fl/fl*^ cerebella, there were almost no S100 positive cells detectable and those that were present did not have any radial processes (Fig. 3t, y).

Finally, we investigated the mice for the development of disease-related symptoms for half a year (Supplementary Fig. 1b). Fifteen out of 33 animals with a homozygous loss of *Brg1* had to be sacrificed in the first 2 weeks of life, indicating that the overexpression of *MYCN* did not compensate for the loss of *Brg1*. In the few animals that survived into adulthood, we did not observe any Brg1 deficient cells in their brains (data not shown). This suggests that in these mice, the simultaneous recombination of the two floxed transgenes (*Brg1* and *lslMYCN*) was not efficient, causing long-term survival. Of note, none of these mice developed a brain tumor. However, some *hG::B*^*fl/wt*^*::N*^*fl/wt*^, *hG::B*^*fl/wt*^*::N*^*fl/fl*^ and *hG::B*^*fl/fl*^*::N*^*fl/wt*^ mice died due to neuroendocrine tumors of the pancreas and the pituitary gland as already reported for *hGFAP-cre::lslMYCN* mice (data not shown) [28]. Hence, the development of these tumors was likely caused by the amplification of *MYCN* but not by the *Brg1* deficiency.

In summary, we did not observe any cooperative effects of Brg1 loss and *MYCN* amplification in these mice, as *hG::B*^*fl/wt*^*::N*^*fl/wt*^ and *hG::B*^*fl/wt*^*::N*^*fl/fl*^ mice developed similar phenotypes as *hGFAP-cre::lslMYCN* mice. Likewise, *hG::B*^*flfl*^*::N*^*fl/wt*^ and *hG::B*^*flfl*^*::N*^*fl/fl*^ mice developed morphological alterations in the brain, which resembled the abnormalities observed in *hGFAP-cre:.Brg1*^*fl/fl*^ mice.

## Discussion

Mutations in *BRG1* and amplifications of *MYCN* have frequently been reported in MBs, especially those of the non-WNT/non-SHH subgroup. This subgroup comprises two thirds of all MBs, but the molecular mechanisms driving oncogenesis are insufficiently understood [4–6, 19, 39]. Therefore, the aim of this study was to elucidate, whether the combination of *Brg1* loss and *MYCN* overexpression in postnatal GNPCs or multipotent NSCs in mice could model the development of human non-WNT/non-SHH MB. To this end, we used *Mert::B::N* and *hG::B::N* mice, and examined histological alterations and survival of these animals.

First, we studied the short-term effects of a postnatal *Brg1* loss and *MYCN* amplification on cell survival parameters of the CGNPs in the EGL of *Mert::B::N* mice. At P8, *MYCN* overexpression in a *Brg1* deprived EGL caused a significant increase in both, proliferating and apoptotic cells. Normal cerebellar development is a highly orchestrated sequence of proliferation, differentiation, migration and programmed cell death. The latter is important to eliminate supernumerary cells from the developing brain (reviewed by [40]). Hence, the increase in cl. Casp3 expressing cells indicating an increase of apoptotic cells in the EGL might just be a compensatory mechanism for the enhancement of proliferation caused by *Brg1* loss and *MYCN* overexpression. *MYCN* is known to be essential for SHH-mediated CGNP proliferation and is amplified and upregulated in human and murine MB, respectively [21, 24, 41, 42]. Previous results already suggested that the enhancement in proliferation caused by *MYCN* amplification alone does not result in MB formation from CGNPs [28]. Even though the postnatal loss of *Brg1* alone appeared to show a trend towards enhanced proliferation, the combination of *MYCN* overexpression and *Brg1* loss did not synergize and did still not result in tumor formation. Nonetheless, we cannot completely preclude that knockdown of *Brg1* and overexpression of *MYCN* might not be fully parallelized events as it might take longer/shorter to accumulate MYCN protein than to lose the Brg1 protein.

The finding of a slightly increased (although not significant) EGL proliferation in *Mert::B*^*fl/fl*^ mice after application of tamoxifen at P3 is still noteworthy, since it contradicts previous results from (non-inducible) *Math1-cre::Brg1*^*fl/fl*^ mice, in which the ATPase is lost at around E10.5. The latter suffer from a severely hypoplastic cerebellum caused by decreased proliferation of GNPCs [16]. Similarly, Zhan et al. reported that loss of *Brg1* at P0 in *Nestin-creER*^*T2*^*::Brg1*^*fl/fl*^ mice resulted in smaller cerebella [43]. Together, in combination with these published data, our here presented results highlight the time-specific role of *Brg1* in the developing cerebellum, a phenomenon that has also been described for other major players in cerebellar development [44, 45].

In addition to the changes in proliferation and apoptosis at P8, we recognized that the induced *Brg1* deficiency in combination with *MYCN* amplification at P3 resulted in the accumulation of cells in the ML at P21. During murine cerebellar development, CGNPs in the EGL first stop proliferating, then start to differentiate and finally migrate towards the IGL, where they start to form synaptic connections [46]. Quantification of Pax6 positive cells revealed that *Brg1* loss by itself or in combination with *MYCN* amplification caused a significant increase in the proportion of granule cells in the ML. This indicates that the migratory behavior of granule neurons was impaired in *Mert::B*^*fl/fl*^, *Mert::B*^*fl/fl*^*::N*^*fl/wt*^ and *Mert::B*^*fl/fl*^*::N*^*fl/fl*^ mice. Of note, many of the cells in the ML were negative for Brg1 in IHC, supporting the hypothesis that the genetic alterations in our mutant mice were directly responsible for the observed phenotype. Likewise, *Brg1* deprivation in cerebellar explants from *hGFAP-cre::Brg1*^*fl/fl*^ mice lead to decreased migration of late-migrating cells in vitro [17]. Additionally, in *hG::B*^*flfl*^*::N*^*fl/wt*^ and *hG::B*^*flfl*^*::N*^*fl/fl*^ mice, the number and morphology of Bergmann glia was severely diminished, indicating that the observed lack of layering in these mice, might be partially explained by disturbed migration as well. Our results suggest that the addition of *MYCN* overexpression enhanced the migration deficit caused by the *Brg1* deficiency. *MYCN* is essential for normal cerebellar development as indicated by different mouse models examining *MYCN* knockout [23, 47]. However, to our knowledge the need for balanced *MYCN* levels for granule cell migration has not been described before. In the adult mice, i.e. 6 months or older, there were no misplaced Pax6 positive cells left in the ML, indicating that granule cell migration was delayed but not permanently inhibited in our model.

A fraction of *hG::B::N* mice developed neuroendocrine tumors of the pancreas and the pituitary gland as previously published for *hGFAP-cre::lslMYCN* mice [28]. Consequently, in these mice, the amplification of *MYCN* determined the phenotype, whereas the heterozygous *Brg1* loss (or the incomplete recombination in mice with a homozygous *Brg1* loss) had no obvious impact. Vice versa, *hG::B*^*flfl*^*::N*^*fl/wt*^ and *hG::B*^*flfl*^*::N*^*fl/fl*^ mice presented with symptoms resembling those of *hGFAP-cre::Brg1*^*fl/fl*^ mice, including early postnatal death, hydrocephalus and hypoplasia of the cerebrum and cerebellum [17]. In these mice, the effect of the homozygous *Brg1* deficiency was dominant and the additional overexpression of *MYCN* did not seem to have an impact. However, as we did not detect MYCN protein expression by IHC in the majority of cerebellar cells at P8, we cannot rule out that the recombination efficiency of the loxP sites of the *lslMYCN* transgene was decreased in case of the additional presence of a homozygously floxed *Brg1* gene. Another more likely explanation for the lack of *MYCN* expressing cells might be that most of the cells hit by the loss of *Brg1* and *MYCN* overexpression have not at all developed or have died by P8. Most importantly, *Brg1* deficiency in combination with overexpression of *MYCN* in *hGFAP* positive NSCs does not result in brain tumor formation.

## Conclusion

In this study, we demonstrate that the combination of a *Brg1* deficiency with *MYCN* amplification in mice is not sufficient to drive tumor formation, neither in postnatal *Math1* expressing cells nor in *hGFAP* positive multipotent NSCs. Consequently, either these cells do not represent cells of origin for non-WNT/non-SHH MBs or the combination of *Brg1* loss and *MYCN* amplification does not provide the genetic basis for tumorigenesis. Still, the animals presented with minor developmental disturbances. Furthermore, in *Math1* positive cells, the combination of the two genetic events seems to have additive effects regarding survival and migration of granule neurons. In contrast, the homozygous knockout of *Brg1* defined the phenotype in *hG::B*^*flfl*^*::N*^*fl/wt*^ and *hG::B*^*flfl*^*::N*^*fl/fl*^ mice, whereas the addition of *MYCN* amplification had no obvious additional effect.

## Supplementary Information

Supplementary Fig. 1 Kaplan-Meier curves of *Mert::B*^*fl/fl*^*::N*^*fl/wt*^, *Mert::B*^*fl/fl*^*::N*^*fl/fl*^, *hG::B*^*fl/wt*^*::N*^*fl/wt*^, *hG::B*^*fl/wt*^*::N*^*fl/fl*^, *hG::B*^*flfl*^*::N*^*fl/wt*^ and *hG::B*^*flfl*^*::N*^*fl/fl*^ mice. Kaplan-Meier curves from P30 onwards of *Mert::B*^*fl/fl*^*::N*^*fl/wt*^ mice (brown) and *Mert::B*^*fl/fl*^*::N*^*fl/fl*^ (purple) shows the decreased survival compared to respective controls (black) in A. However, none of the animals developed a tumor. The survival curves of *hG::B*^*fl/wt*^*::N*^*fl/wt*^ (blue), *hG::B*^*fl/wt*^*::N*^*fl/fl*^ (orange), *hG::B*^*flfl*^*::N*^*fl/wt*^ (green) and *hG::B*^*flfl*^*::N*^*fl/fl*^ mice (red) show diminished survival compared to respective controls (black) and are depicted in B. Still, none of the animals developed a brain tumor. ^**^p < 0.01, ^***^p < 0.001, ^****^p < 0.0001. n.s., not significant. (PNG 51.4 kb).

High resolution (51.4 kb).
