# Supplementary figures and images for: Simultaneous Brg1 Knockout and MYCN Overexpression in Cerebellar Granule Neuron Precursors Is Insufficient to Drive Tumor Formation but Temporarily Enhances their Proliferation and Delays their Migration
Source: Cerebellum. 2021 Jan 2;20(3):410–9. doi: 10.1007/s12311-020-01219-2 (PMC8213679; doi:10.1007/s12311-020-01219-2)

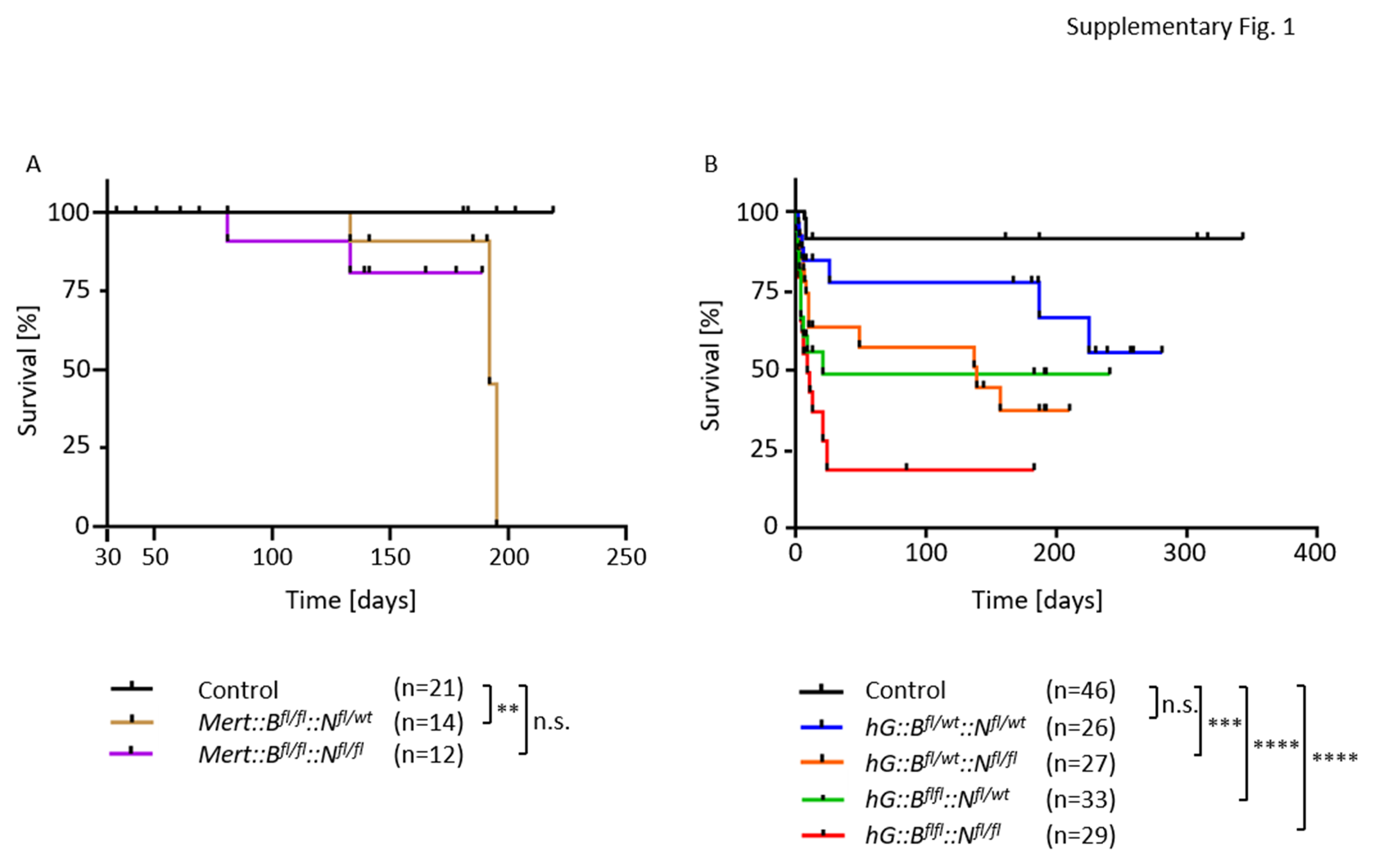

Supplement: Supplementary file 1 — Kaplan-Meier curves of Mert::Bfl/fl::Nfl/wt, Mert::Bfl/fl::Nfl/fl, hG::Bfl/wt::Nfl/wt, hG::Bfl/wt::Nfl/fl, hG::Bflfl::Nfl/wt and hG::Bflfl::Nfl/fl mice. Kaplan-Meier curves from P30 onwards of Mert::Bfl/fl::Nfl/wt mice (brown) and Mert::Bfl/fl::Nfl/fl (purple) shows the decreased survival compared to respective controls (black) in A. However, none of the animals developed a tumor. The survival curves of hG::Bfl/wt::Nfl/wt (blue), hG::Bfl/wt::Nfl/fl (orange), hG::Bflfl::Nfl/wt (green) and hG::Bflfl::Nfl/fl mice (red) show diminished survival compared to respective controls (black) and are depicted in B. Still, none of the animals developed a brain tumor. **p < 0.01, ***p < 0.001, ****p < 0.0001. n.s., not significant. (PNG 51.4 kb). [file 12311_2020_1219_Fig4_ESM.png]

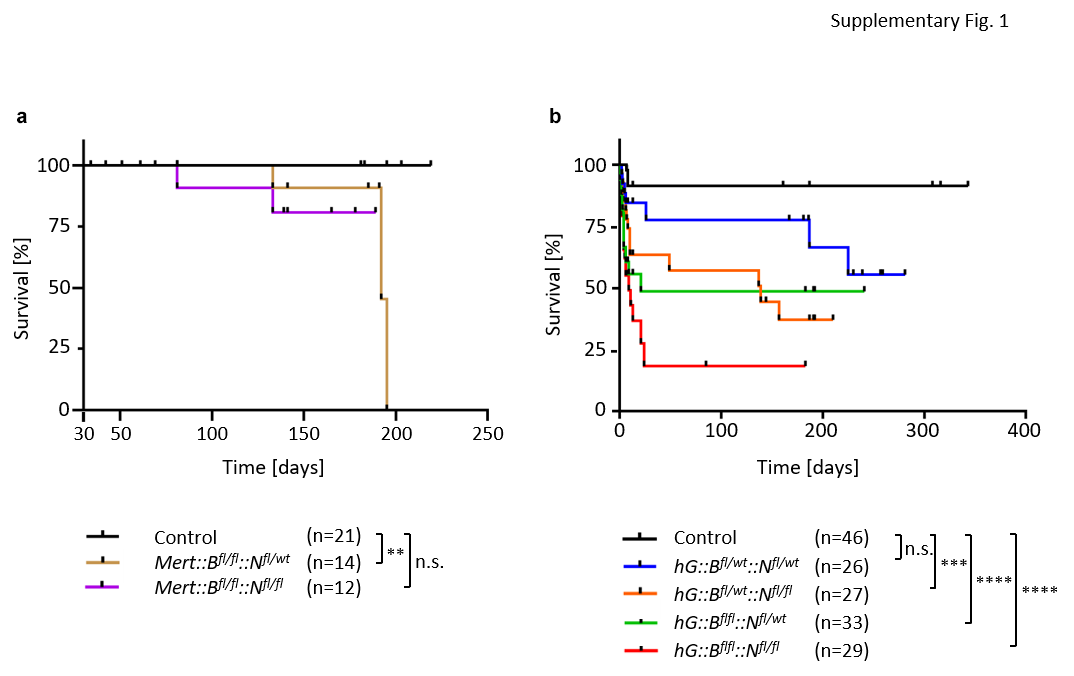

Supplement: Supplementary file 2 — High resolution (51.4 kb). [file 12311_2020_1219_MOESM1_ESM.png]
